# DEVELOPMENT OF AN ONLINE DISASTER PREPAREDNESS PROGRAM FOR CAREGIVERS OF PERSONS WITH DEMENTIA

**DOI:** 10.1093/geroni/igad104.2566

**Published:** 2023-12-21

**Authors:** Maria Donohoe, Nicholas Ostrem, Nicholas Rudzianski, Jane Gay, Brigid Smith, Tiffany Wunderlich, Sato Ashida

**Affiliations:** University of Iowa College of Public Health, Iowa City, Iowa, United States; University of Iowa College of Public Health, Iowa City, Iowa, United States; University of Iowa College of Public Health, Iowa City, Iowa, United States; University of Iowa College of Public Health, Iowa City, Iowa, United States; University of Iowa College of Public Health, Iowa City, Iowa, United States; University of Iowa College of Public Health, Iowa City, Iowa, United States; University of Iowa College of Public Health, Iowa City, Iowa, United States

## Abstract

Individuals with medical conditions, including persons with dementia (PWD), are vulnerable in disaster situations. Disasters limit caregivers’ ability to continue with care due to stress, reduced resources and limited social support. We previously developed and tested a disaster preparedness program for older adults, Disaster PrepWise, and showed its impact on improving preparedness and personal emergency support networks. Through community engaged approaches, we adopted this program to specifically support family caregivers of PWD. Contents were revised based on the emergency management and dementia caregiving literature, and input from the stakeholder advisory board (SAB) consisting of caregivers, aging service providers, public health, and emergency management agencies. The SAB also provided guidance on implementation strategies from the lens of over-burdened caregivers. The new program includes a five-step process with an emphasis on the needs of caregivers and caregiving tasks, for example, developing emergency caregiving network to be activated during and after a disaster. The program also informs caregivers of tasks involved in the event of an emergency and provides various information such as how to navigate staying at public shelters with PWD, identify services during and after an emergency, and manage stress. Preliminary data shows high levels of acceptance and perceived usefulness of this caregiver-specific program. The program is perceived to fill a gap in current disaster management approaches that have limited ability to support vulnerable populations. Caregivers and service providers also praised this program’s ability to connect caregivers to community programs and resources. Additional pilot data will be presented.

